# Open craniofacial reconstruction for coronal craniosynostosis

**DOI:** 10.3171/2021.1.FOCVID20122

**Published:** 2021-04-01

**Authors:** Edward R. Bader, Adam Ammar, Adisson N. Fortunel, Rafael De la Garza Ramos, Oren Tepper, Andrew J. Kobets

**Affiliations:** 1 Department of Neurological Surgery and; 2 Division of Plastic and Reconstructive Surgery, Montefiore Medical Center, Bronx, New York

**Keywords:** craniofacial reconstruction, craniosynostosis, cranial vault remodeling, pediatric neurosurgery

## Abstract

Here the authors demonstrate open craniofacial reconstruction for the correction of craniosynostosis, using techniques refined by Dr. James T. Goodrich at Montefiore Medical Center. They present the operative management of a case of unilateral coronal synostosis in a 12-month-old child, who presented with right forehead prominence and calvarial asymmetry. The patient had an excellent correction of her head shape with an uneventful postoperative course. This video highlights the authors’ multidisciplinary approach to complete cranial vault remodeling, utilizing a Marchac bandeau construct and split calvarial graft mosaic technique.

The video can be found here: https://vimeo.com/519489422.

## Transcript

Here we present craniofacial reconstruction for craniosynostosis, the open approach that is used at Montefiore Medical Center. Our approach is an open complete cranial vault remodeling, of which the late Dr. James T. Goodrich was an avid proponent. We favor this approach as opposed to less invasive techniques in older children in order to achieve the best cosmetic outcomes for our patients, in our opinion. We prefer to perform our cranial vault reconstructions as a multidisciplinary effort with our plastic surgery colleagues for the best results for our patients. This allows us to combine our expertise as well as to expedite the operation as we work in conjunction in the operating room.

**1:01 Case History**. Our patient today is a 12-month-old female who originally presented to our clinic with a misshapen head. On examination, the patient was found to have asymmetrical orbits, with right forehead prominence and calvarial asymmetry, leading to the diagnosis of left coronal synostosis. Our diagnosis was confirmed with CT imaging and the patient was scheduled for a craniofacial reconstruction to correct the defect.

**1:25 Preoperative Imaging**. As you can see here, CT reconstructions demonstrate early closure of the left coronal suture, flattening of the left frontal and parietal bones, and the subsequent harlequin eye.

**1:38 Positioning and Marking**. Positioning in the operating room consists of utilization of a padded horseshoe head holder in order to support the patient's head. In anterior cranial vault remodeling, which this patient will undergo, the patient is placed supine with exposure of the ears bilaterally, as well as the orbits, in order to expose the entire operative field from the orbital roof down to the ears bilaterally and as far back as 2 cm behind the coronal suture. A strip of hair is shaved 1 cm anterior to the coronal suture, and the incision line is drawn. We utilize a lazy-S incision as we believe that helps with skin relaxation and assists with closure at the end of the case. In this patient, the incision was placed more anterior than normal due to the shape of the patient's head in order to allow us to reach the orbital roof. The patient is prepped and draped in a sterile fashion; we prefer a povidone-iodine solution.

**2:37 Scalp Dissection**. The skin is incised with careful consideration of hemostasis in young children, utilizing monopolar and bipolar cautery to achieve it. The skin incision is carried from ear to ear, and down to the pericranium, taking care not to violate it. The skin is then elevated anteriorly along the subgaleal layer. The pericranium is then incised 2 cm either posterior or anterior to the incision and carried to the temporalis muscle attachments, then elevated anteriorly, taking care for proper hemostasis using ample bone wax, until the calvaria is exposed.

**3:14 Craniotomy**. A Marchac template is then used in the right frontal region to map cuts for the neo-forehead that will be used for reconstruction. The bone graft will be harvested using the entire anterior calvaria down to the orbital roofs. Here you see painting of that template and the cuts that will be taken. Burr holes are made with an M33 or Acorn drill bit outside of the template lines in order to reduce the size of the neo-forehead. Ample bone wax and FLOSEAL is used to ensure proper hemostasis. Burr holes are created, and the dura carefully separated from the bone. A B5 drill bit is used to cut out the template with care not to violate the dura and especially the sinus medially. We like to use a special suction to collect the bone dust from our drilling for use later as we reconstruct the cranial vault. Once all cuts are made, the Marchac template is elevated and removed with care not to violate the dura. Here you see the elevation of that Marchac template and no dural defects. The rest of the calvaria is then separated from the dura using a combination of FLOSEAL and elevators, and then drilled and removed in a similar fashion. Here we see the elevation of the calvaria with the Marchac template and then retraction of the brain to expose the orbital roof. The next step will be the elevation of the orbital roof utilizing a bandeau cut, as has been described previously in the literature.^1^

**5:10 Orbital Roof Elevation**. The zygomaticofrontal and temporal bones are then cut with care to protect the dura and brain. In our case, dissection around the sphenoid wing was achieved with relative ease; however, due to the heterogeneity of unicoronal synostosis, in some cases the dissection around the sphenoid wing can be difficult. As the nasofrontal junction is cut, the orbits are retracted slightly. It is important to communicate continuously with the anesthesia team during this part of the procedure as depression of the orbits can cause bradycardia. If this occurs, retraction should be stopped immediately, and the procedure stopped until the bradycardia resolves. The final cuts are made along the orbital roofs until the bandeau is complete and the orbital roof can be easily elevated. Here, we see the orbital roof elevated easily. As you can see, no dural defects have been made.

**6:03 Neo-Forehead Construction**. The neo-forehead from the Marchac template is then bent to form an appropriate curve for the new forehead and calvaria. The bandeau cut is also bent to match this curve. The rest of the harvested calvaria is then split, and one long longitudinal piece is cut to be used to cover the sagittal sinus and be used as part of the frame for the new calvaria. The rest of the bone graft is broken into smaller pieces that will be used to recreate the calvaria. The neo-forehead is then attached to the bandeau using absorbable plates to create the new forehead for the patient, as seen here. Here you see the new forehead, which is then returned to the orbital roof and secured in place with absorbable plates.

**6:54 Calvarial Reconstruction**. As this is done, the temporal muscle on the synostotic side needs to be moved forward along with the bone. An overcorrection on the synostotic side should be done to avoid relapses with this technique. As you see, this provides ample space for the growth of the brain and has a cosmetically pleasing appearance. The longitudinal piece is placed over the sagittal sinus and secured with absorbable plates. Then multiple holes are drilled into the bone for PDS sutures to be run through for the creation of the lattice work. The PDS sutures are run anterior-posteriorly and then medial-laterally, intertwining the sutures to create a lattice frame to hold the harvested bone grafts. The lattice should be tight enough to hold the bone fragments, but not so tight as to compress the brain. Care is taken not to puncture the dura as CSF leak at any point during the procedure greatly increases the risk of postoperative complications. The bone fragments are slid in place to form a mosaic to cover the dura. Here you see the finished calvaria with a cosmetically pleasing shape and full coverage of the dura.

**8:10 Scalp Closure**. The next step is important, as closure begins with the pericranium that is stretched back over the bone and secured with 3-0 Vicryl sutures. This is important as the pericranium contains the vascular supply for the bone and helps to reduce bone resorption; it is important that as much of the bone as possible is covered with the pericranium as seen here. The skin is then closed in two layers, with 3-0 Vicryls used for the galea and a running absorbable suture such as Monocryl or Rapide for the superficial skin.

**8:42 Postoperative Course**. Here you see an excellent result with good head shape. Postoperatively, the patient had an uneventful course and was observed in the ICU for one night, as is our usual protocol. We also provide our patients with 48 hours of postoperative prophylactic antibiotics and observe them in the hospital for approximately 4 days. In this particular case, the patient was discharged home on postoperative day 5. We maintain a clean head wrap over the wound until the postoperative clinic appointment, usually 1–2 weeks after surgery.

**9:17 Follow-Up**. On follow-up at 3 months, the incision was well healed and the patient had an excellent correction of her head shape.

**9:28 Tribute to James T. Goodrich**. We would like to dedicate this video to the late Dr. James Tait Goodrich, who was a master of the craniofacial reconstruction and was an excellent mentor.

## Author Contributions

Primary surgeon: Tepper, Kobets. Assistant surgeon: De la Garza Ramos. Editing and drafting the video and abstract: Bader, Ammar, Fortunel. Critically revising the work: Bader, Ammar, Fortunel, De la Garza Ramos, Kobets. Reviewed submitted version of the work: Bader, Ammar, Fortunel, De la Garza Ramos, Kobets. Approved the final version of the work on behalf of all authors: Bader. Supervision: Kobets.

## References

[1] Lopez CD Kumar A Lin AY. Demystifying the “triple point”: technical nuances of the fronto-orbital advancement J Craniofac Surg 2018 29 3 796 799 29489569 10.1097/SCS.0000000000004147

